# Prewetting Electron Beam Irradiated and Native Sheep Wool Can Affect Their Sorptivity

**DOI:** 10.3390/polym15214267

**Published:** 2023-10-30

**Authors:** Karin Koóšová, Jana Braniša, Andrej Dubec, Mária Porubská

**Affiliations:** 1 Department of Chemistry, Faculty of Natural Sciences and Informatics, Constantine the Philosopher University in Nitra, Tr. A. Hlinku 1, 949 74 Nitra, Slovakia; karin.koosova@ukf.sk (K.K.); jbranisa@ukf.sk (J.B.); 2 Department of Materials Engineering, Faculty of Industrial Technologies, Alexander Dubček University of Trenčín, I. Krasku 491/30, 020 01 Púchov, Slovakia; andrej.dubec@tnuni.sk

**Keywords:** sheep wool, electron irradiation, prewetting, adsorption, complexing effect

## Abstract

In this work, the effect of prewetting native and electron beam-modified wool on the resulting sorption of Cu(II) from wool solutions was studied. The following conditions and combinations were applied: 6 mM and 50 mM solutions, prewetting time 0–24 h, contact time 1–24 h. The sorption results showed that wetting the wool before sorption from the low concentrated solution can fundamentally improve the efficiency of the separation process. The opposite result was achieved when applying a more concentrated solution; that is, prewetting slightly reduced the sorptivity. The reasons for such results are discussed. The application of these findings can be used to optimize the separation process in technological practice, however, will require solute specification.

## 1. Introduction

The meaningful application of renewable material waste contributes to reducing the carbon footprint. Such waste includes sheep wool that is unsuitable for textile applications. Indeed, the disposal of woolen waste, classified as hazardous waste of animal origin, is complex and costly.

Keratin—an essential wool component—comprises a protein-type biopolymer with side chains containing acidic and basic functional groups, which provide a prerequisite for good adsorption properties [1,2]. Researchers have aimed to improve the adsorption capacity of wool via chemical modifications involving the introduction of new functional groups to increase the number of binding points [3,4,5,6,7,8,9]. However, each chemical process is associated with the use of chemicals and wastewater production. Meanwhile, we recently described an original wool modification process that avoids these negative effects by irradiating wool with an accelerated electron beam [10]. This simple, time-saving, and dry technological process permanently affects the entire volume of wool fiber. However, the resulting properties of such modified material remain to be fully characterized. Our previous studies [11,12] suggest improved adsorption, which can be exploited in numerous technological processes.

Adsorption separation techniques are modified according to the purpose of use and the nature of the adsorbent and separated components. Regarding adsorbents, they can be characterized by the percentage of removal/recovery of the monitored component of the solution under specified conditions or by the required contact time of the solute with the adsorbent to achieve optimal sorptivity. In the latter parameter, individual adsorbents can vary considerably.

Contact times from 5 min to 24 h were applied in a comparative study on the adsorption of six divalent heavy metal cations on a MnFe_2_O_4_@CAC hybrid adsorbent [13]. Meanwhile, Mongioví et al. [14] report a 15-min agitation method for removing metal using hemp and flax fiber. Tran et al. [15] studied heavy metal biosorption from aqueous solutions by algae inhabiting rice paddies, applying contact times ranging from 30 min to 12 h. Shaking for 40 min was used to evaluate copper adsorption from wastewater on green algae cultured with *L-cysteine* [16]. Moreover, adsorption of chromium(VI) from wastewater on agro and horticultural adsorbents occurs within 45 min [17]. Adsorption of heavy metals on synthesized gelatin-based nanocomposite biosorbent requires stirring of the components for 60 min [18]. Adsorbent ultrasonically modified chitin was agitated for 6 h during the adsorption of a ternary system containing cobalt, nickel, and methylene blue [19]. However, longer contact times have also been reported. For instance, a 24-h agitation was applied for the multi-component competitive adsorption of heavy metal ions on raw and acid-treated bush mango and flamboyant biomasses [20]. Similarly, stirring for 24 h was required when a pyrolyzed flamboyant biomass-based adsorbent in the form of lignocellulosic char was combined with Pb(II), Hg(II) and Zn(II) [21].

Fewer data describe adsorption with sheep wool as a representative material of animal origin. The specificity of wool as a keratin material is based on its structure, comprising basic and acidic groups, the proportion of which changes with the pH of the solution. Keratin has several supramolecular structures that become altered with temperature, humidity, and mechanical stress. Indeed, wool is a well-suited bioadsorbent and is a renewable material. This was demonstrated by Dakiky et al., who employed sheep wool to remove chromium(VI) from industrial wastewater with contact times that varied from 30 to 300 min [22]. Similarly, Wen et al. examined the removal of Co(II) from aqueous solution using wool powder [23] with contact times of 15 min–22 h. Meanwhile, the adsorption of Cu(II), Hg(II), and Ni(II) ions on natural wool fibers grafted with ethyl acrylate requires a 3-h processing time [3]. However, few studies on sheep wool adsorption have described different experimental conditions, including the solution pH, mass-to-solute ratio, tested solute concentrations, and contact time. Hence, comparing optimal contact times between these individual cases is difficult.

Achieving adsorption equilibrium with certain materials is challenging and sometimes impossible. In addition, determining the residual solute concentration during the wool–solute interaction can be affected by various processes, including time-dependent hydrolysis, oxidation-reduction processes, and formation of associates or micro-precipitates. Moreover, in the case of fibrous adsorbents, the disintegration of the fiber (abrasion, cracking of the surface layer, fragments) may occur during the shaking process. Additionally, the imperceptible turbidity associated with UV-VIS spectral analysis of filtrates will distort the experimental results. It is, therefore, essential to determine the optimal duration and nature of the contact between the adsorbent and solute on the one hand and the acceptable adsorption equilibrium on the other.

Our previous studies assessed the adsorption of metal cations on natural and electron-irradiated wool, revealing that the estimated optimal contact time for the wool and solute was 24 h; that is, 6 h of shaking and 18 h of static mode [11,12]. However, the effort required to reduce the contact time while achieving maximum adsorption prompted us to delve deeper into this issue in the current study. 

## 2. Experimental

### 2.1. Materials

Copper(II) sulfate pentahydrate (CuSO_4_·5H_2_O) of analytical grade with spectral λ_max_ = 809 nm was supplied by Centralchem (Bratislava, Slovakia) and used as sorbate. Surfactants Lansurf OA7 (nonionic surfactant produced by the alkoxylation of ethylene oxide with oleic acid) and Lansurf AE107 (nonionic surfactant produced by the reaction of 7 moles of ethylene oxide onto a 2-propyl heptanol base) were supplied by Lankem Ltd. (Dukinfield, Cheshire, UK) and used to measure surface tension of wool fibers. Sheep wool came from spring sheep-shearing of a Tsigai-Suffolk crossbreed bred in Middle Slovakia. The wool samples were randomly collected from various sites, and the fiber thickness ranged from 27 to 33 μm.

### 2.2. Sheep Wool Scouring

After manually removing the wool’s crude impurities, the wool was scoured repeatedly in warmish tap water until the rinse water was clean. The 5-L ultrasonic bath Kraintek 5LE (Podhájska, Slovakia) was used for scouring. Approximately 20 g of wool was placed in the bath and washed in 40 °C tap water for a 10 min period. The water was then changed, and the washing was repeated. Finally, the wool was rinsed with 5 L of demineralized water. After the trapped water ran off, the sample was air-dried in the laboratory for 48 h and stored under standard laboratory conditions.

### 2.3. Sheep Wool Irradiation

The samples were placed in separate unsealed polyethylene pouches in carton boxes and irradiated in a linear electron accelerator UELR-5-1S (FGUP NIIEFA, Petersburg, Russia) operated by Progresa Final SK (Bratislava, Slovakia). The process parameters were as follows: installed energy of 5 MeV, intensity of 200 μA, mean power of 1 kW, and mean dose rate of 750 kGy/h. The doses applied were 0, 48, and 153 kGy, which were checked dosimetrically. Samples were then stored under standard laboratory conditions.

### 2.4. Electron Microscopy

The fiber samples were cleaned in isopropyl alcohol and dried with nitrogen. Cross-cutting was performed with a sharp cutting tool at −18 °C. The Quorum SC7620 Sputter Coater (Quorum Technologies, Laughton, East Sussex, UK) was used to create an Au-Pd conductive layer on the surface. Scanning Electron Microscopy (SEM) images of the wool fibers were captured by an electron microscope (TESCAN Vega 3; Brno, Czech Republic) operating in the secondary electron mode.

### 2.5. Measurement of Wool Surface Tension

The surface tension of wool fibers was determined using the flotation method [24]. A wool fiber snip < 0.5 cm in length was placed into a 0.1% solution of Lansurf OA7 and titrated with a 0.1% solution of Lansurf AE107. The surface tension of the titrated solution with the floating snip was measured with a stalagmometer and identified via fiber surface tension.

### 2.6. Stalagmometric Measurement of Surface Tension of Liquids

The surface tension of water $\gamma_{H_{2}O}$ and Cu-solutions $\gamma_{\mathrm{Cu}\left(\mathrm{II}\right)}$ was estimated using a Traube stalagmometer [25] by weighing 20 drops with 9 repetitions. Surface tension $\gamma_{H_{2}O}$ was calculated using Equation (1):

(1) $$\gamma_{H_{2}O}=\left(75.872-0.154\;\cdot \;t-0.00022\;\cdot \;t^{2}\;\right)\cdot 10^{-3}$$

where $\gamma_{H_{2}O}$ is surface tension of water (mN/m) and t is temperature (°C) of H_2_O (standard).

The surface tension of the Cu(II) solutions $\gamma_{\mathrm{Cu}\left(\mathrm{II}\right)}$ was calculated according to Equation (2):

(2) $$\frac{\gamma_{\mathrm{Cu}\left(\mathrm{II}\right)}}{\gamma_{H_{2}O}}=\frac{m}{m_{H_{2}O}}$$

where m is the mass of a certain number of Cu-solution drops in g, and $m_{H_{2}O}$ is the mass of the same number of water (standard) drops in g.

### 2.7. Batch Sorption Experiments

Under sorption experiments with non-prewetted wool, 40 mL of 6 mM or 50 mM Cu(II) test solution was added to 0.2 g of cut wool. In the case of 24 h contact, the mixture was shaken (Shaker Witeg SHR-2D, Labortechnik GmbH, Wertheim, Germany) for 6 h and left in static mode for 18 h. Shorter contact periods were achieved by shaking for 6 h and completing the period in static mode.

To perform the prewetting process, a 0.2 g cut wool sample was immersed in 20 mL of deionized water and left in static mode for the specified time. Then, 20 mL of 12 mM or 0.1 M test solution was added so that the final Cu(II) concentration and sorbent/solute ratio were the same in the non-prewetted and prewetted samples. The shaking/static contact mode was the same as that of the corresponding non-prewetted samples. The mixture was filtered through KA4 filter paper, and the filtrate was used to determine the residual Cu(II). Each sorption experiment was performed in triplicate.

### 2.8. Determination of Residual Cu(II) Concentration

The determination of residual Cu(II) was performed spectroscopically by scanning the VIS spectrum of CuSO_4_ using the Specord 50 Plus Spectrophotometer (Analytikjena, Jena, Germany) and evaluating the absorbance at λ_max_ = 809 nm. The water extract from the corresponding wool sample obtained under identical conditions as the Cu(II) residual solution was used as a blank for each series of samples with identical absorbed doses. For the absorbance of the filtrate to be in the 0.1–1.0 range, a 5 cm cuvette was used for the 6 mM solution and a 1 cm cuvette for the 50 mM solution. Calibration curves were prepared accordingly.

### 2.9. Determination of Sorptivity

The sorptivity, defined as the mass of Cu(II) in mg per 1 g of wool, was estimated using Equation (3):

(3) $$S=\frac{x_{1}-x_{2}}{m}$$

where *S* is the sorptivity for individual wool samples when Cu(II) is applied at the specified concentration, *x*_1_ is the mass of Cu(II) added to the solution (mg), *x*_2_ is the residual mass of Cu(II) in the solution after its contact with the wool sample (mg), and *m* is the mass of the wool sample analyzed (g).

### 2.10. Estimation of Standard Deviation

The Dean-Dixon method [18] was used to estimate the standard deviation *s*. This method is suitable for estimating the standard deviation when a small number of parallel tests (n = 2–9) is applied. Calculation of *s* was performed using Equation (4):

(4) $$s=k_{n}\;\cdot \;R$$

where *k_n_* is the Dean-Dixon’s coefficient (k*_n_*_=3_ = 0.5908), and *R* is the difference between the maximum and minimum partial results in an individual set.

## 3. Results and Discussion

The contact time between the adsorbent and the adsorbate is one of several factors influencing the achievement of maximum sorption. In most cases, it is assumed that increasing the contact time leads to higher sorptivity. However, the degree of influence of the contact time depends on the nature of the adsorbent and adsorbate.

**Figure 1:**
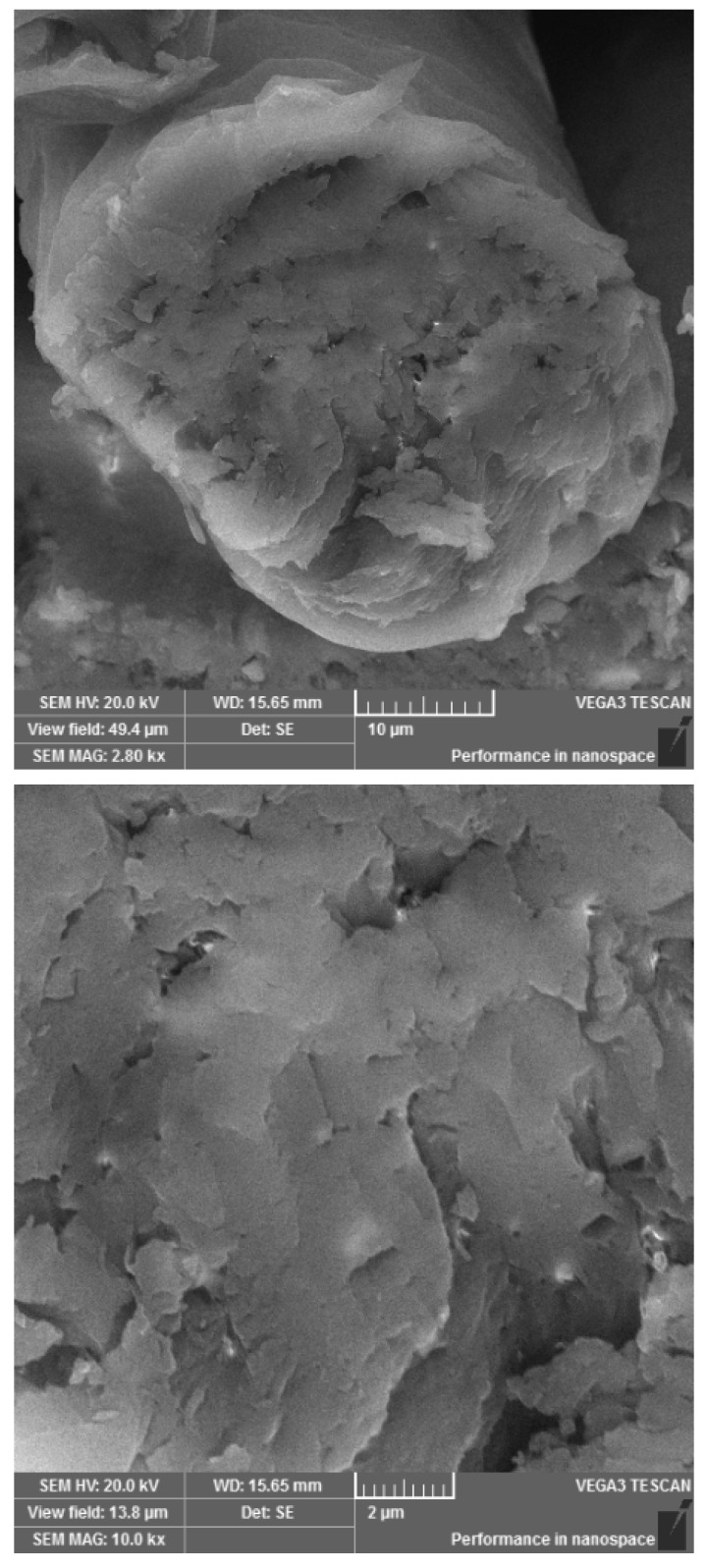
The cross-section of non-irradiated wool fiber under different resolution.

Sheep fiber has a complex structure. The surface of raw wool is covered with water-repellent wax—lanolin. Even if scoured before processing, depending on the technology and chemicals used, some residual bound wax remains on the wool surface. The surface cuticle cells of the fiber protect against fiber damage. Moreover, the cells have a waxy coating, making wool water-repellent while allowing water vapor absorption. The interior of the wool fiber, the cortex, is made up of long cortical tapering cells that overlap. They are surrounded by the cell membrane complex found throughout the fiber, which comprises proteins and waxy lipids. The tapering cells present vacuoles, in which moisture collected from the wetter external environment is collected. A cross-section of a non-irradiated wool fiber is presented in Figure 1.

**Figure 2:**
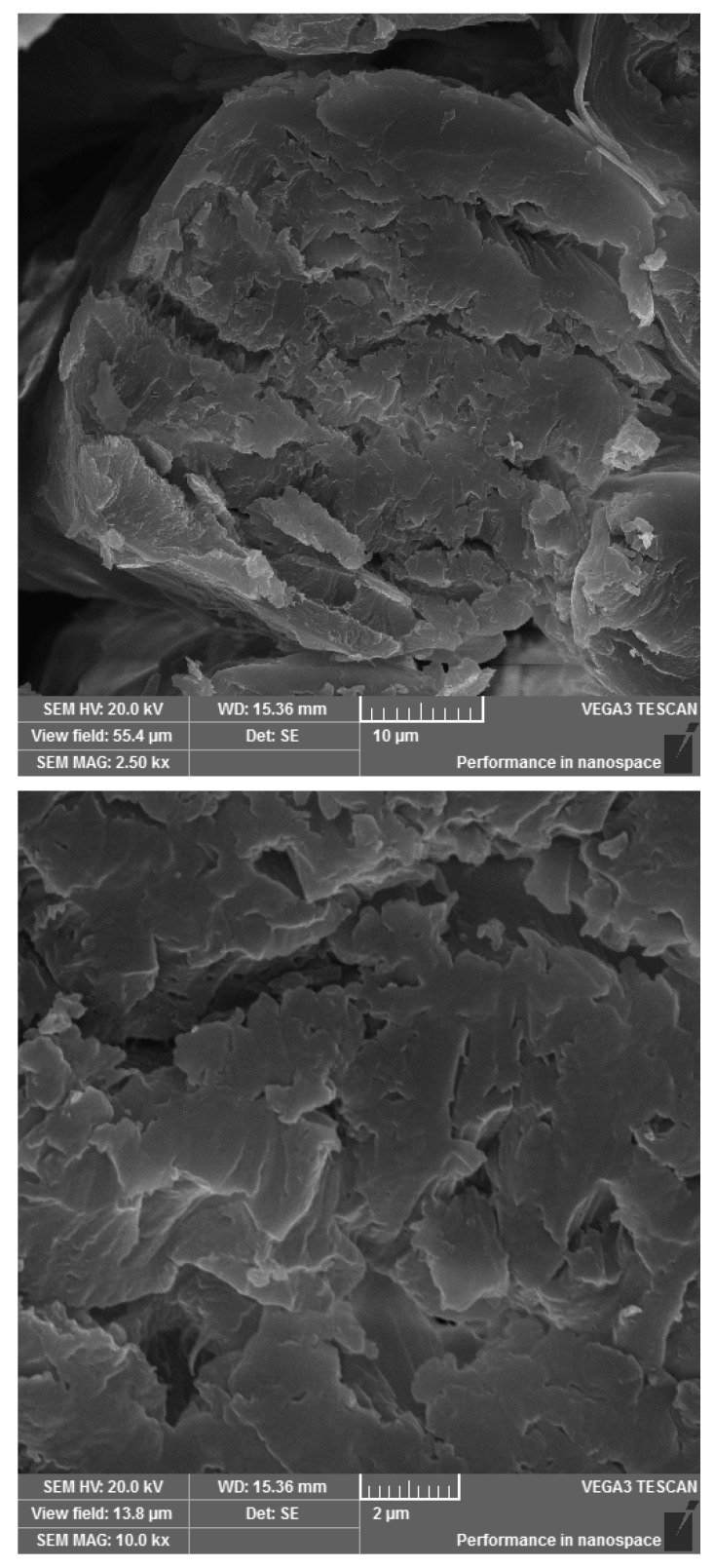
The cross-section of irradiated wool fiber under different resolution.

The energy of the accelerated electron beam changes the chemical and physical structure of the fiber [10]. The key factor is the absorbed energy dose. The change in physical structure is evident in Figure 2, taken from a cross-section of an irradiated fiber with a higher absorbed dose. Figure 1 shows a continuous fiber matrix with isolated pores corresponding to the original closed vacuoles, while in Figure 2, in addition to vacuoles, the matrix continuity is disrupted, and formed cracks and pores are visible.

**Scheme 1:**
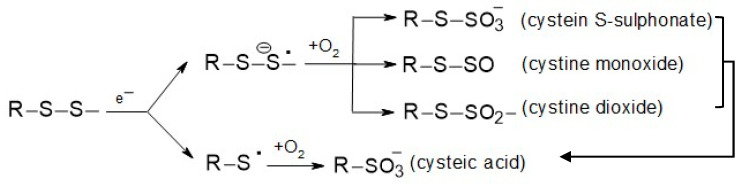
Generation of S-oxidized products in keratin after wool irradiation by an accelerated electron beam.

These modifications result from chemical changes [10], which can be briefly described as follows:

From Scheme 1, it follows that the breaking of disulfide bridges between pairs of keratin chains and the subsequent incorporation of oxygen into the structure to form cysteic S-sulphonate, cystine monoxide, cystine dioxide, and finally, cysteic acid must also have disrupted the phase of the original ordered supramolecular structure. These transformational changes lead to the amorphization of the fiber.

**Figure 3:**
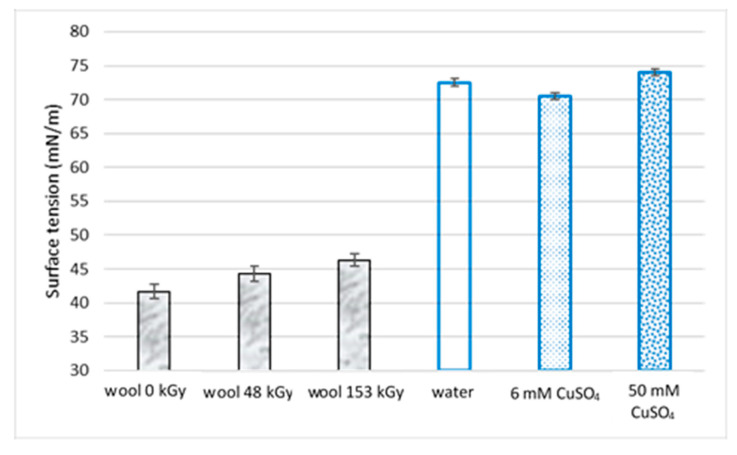
Surface tension of sheep wool and selected liquids.

The presence of waxy substances across the entire fiber indicates the hydrophobicity of the adsorbent. Suppose a woolen fiber is used for adsorption from an aqueous environment. In that case, the hydrophobicity of the fiber is a factor determining its willingness to be wetted by aqueous adsorbate solutions. Wetting the wool is the first stage of the adsorption process and largely determines the adsorption rate. From a thermodynamic point of view, the wetting process is characterized by enthalpy. According to the IUPAC document [26], the enthalpy of wetting Δ_w_H (−Q_w_) or enthalpy of immersion Δ_imm_H (–q_w_), when referring to the unit of mass of the solid, is defined as the difference between the enthalpy of a solid completely immersed in a wetting liquid, and that of the solid and liquid separately. If the purpose is not to directly measure the Δ_w_H of wool, an indicator of the wetting tendency is the relationship between the surface tension of the liquid medium and the adsorbent. As can be seen from Figure 3, the surface tension of the used wool samples and aqueous solutions is significantly different. The measured values of the wool surface tension are similar to those of polymers with poor wettability, such as polyamide 6 (48.3 mN/m), poly(ethylene terephthalate) (44 mN/m), or poly(ethylene) (34 mN/m), while for example, poly(tetrafluoroethylene) has only a half value (23 mN/m) [27].

Irradiation of the wool by electrons affects its structure, including the surface tension (Figure 3) and, accordingly, the wetting process. The resulting S-oxidized structures (Scheme 1) have a higher polarity than the non-irradiated wool and should facilitate the wetting of the exposed wool with water. Our experimental results confirmed this expectation (Figure 3). The increase in polarity only alleviated the difference in the measured surface tension of wool, water, and aqueous Cu-solution samples (Figure 3).

As mentioned above, experimental adsorption times reported in the literature are variable. From the data, it can be concluded that plant-based adsorbents are more easily wetted by water. Plants have a waxy surface, disrupted upon disintegration before use as an adsorbent. However, plant subsurface layers are carbohydrate in nature, with surface tension closer to that of water than wool, so they can distribute water-soluble minerals and assimilate to all plant tissues and organs. Therefore, the time to reach equilibrium adsorption is shorter. Although the authors did not address this aspect, in the case of wool, we hypothesized that if the process of adsorption on wool was preceded by wetting wool with water, the adsorption process could be accelerated. In such a case, the first step is to immerse the adsorbent in a pure solvent and saturate it with water. The second step is contacting the prewetted adsorbent with the solute solution when a solution replaces the pure solvent, and the adsorption of the solute takes place only by the displacement of the solvent. Thus, the solute’s adsorption causes solvent desorption, but not always by an equivalent amount [28]. Due to the small difference in surface tension of water and the solution compared to wool (Figure 3), the water exchange process with the solute should be faster than wetting wool with water. The rate of exchange will depend on the properties of the solvent—solute partners. We tested this hypothesis on Cu(II) adsorption from 6 mM and 50 mM CuSO_4_ solutions.

### 3.1. Testing of Wool Prewetting Using Cu(II) Adsorption from 6 mM-Solution

#### 3.1.1. Non-Prewetted Sheep Wool

**Figure 4:**
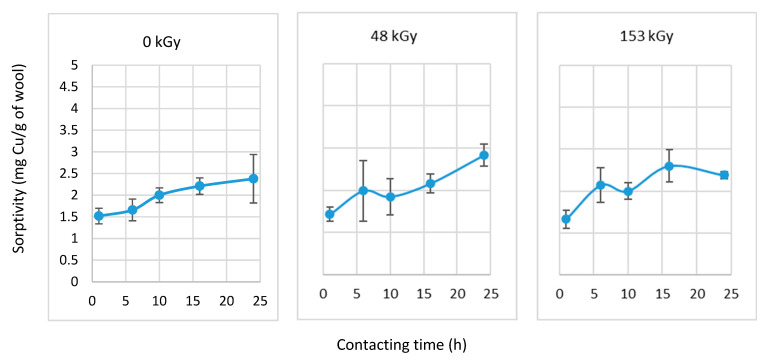
Dependence of sorptivity of non-prewetted wool on contact time with CuSO_4_ solution (6 mM).

Dispersion of partial results for different measured wool parameters is common even with the same breed and age of sheep. This is influenced by the fiber thickness distribution, the sampling site on the sheep, breeding conditions, etc. The effect of the electron beam further increases the assumptions for the variability of partial results. Therefore, it is necessary to consider this when evaluating the data. The sorptivity of wool with a 0–48–153 kGy dose without preliminary wetting was measured after different contact times in the 1–24 h range. The results are shown in Figure 4.

The sorptivity of non-prewetted dry wool from 6 mM Cu(II) solution (Figure 4) increased with contact time. The increase was monotonic in the 0 kGy sample, while the 48 and 153 kGy-dosed samples exhibited a variable course since sorptivity is also a function of the absorbed dose. This was expected as each absorbed dose of energy induces a specific change in keratin and the formation of intermediates or final species. The electron beam modifies keratin’s primary and secondary structure, which impacts the number of active sites for adsorption. Moreover, as evidenced in Figure 1 and Figure 2, the physical conditions for solute diffusion into the fiber matrix also change. Under the applied conditions, the sample with the 48 kGy dose showed the highest sorptivity after 24 h of contact with the Cu(II) solution. Within each data set, it can be generally concluded that increasing the contact time of the wool with the aqueous solution increases the sorptivity.

#### 3.1.2. Prewetted Sheep Wool

**Figure 5:**
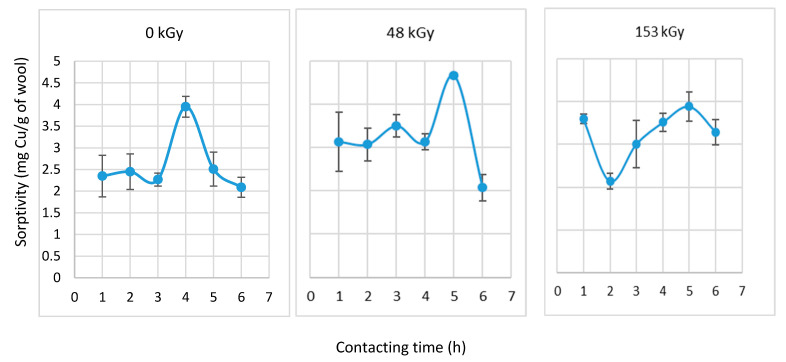
Sorptivity of wool after 24 h of prewetting at selected contact times with CuSO_4_ solution (6 mM).

In another experiment, wool samples were prewetted in a defined volume of water for 24 h. The Cu(II)-solution was then added to the fiber suspension so that the resulting concentration of Cu(II) was 6 mM and the mass-to-solution ratio was the same as for the non-prewetted wool. After the selected shaking interval (1–6 h), the samples were processed, and the sorptivity was calculated (Figure 5).

**Figure 6:**
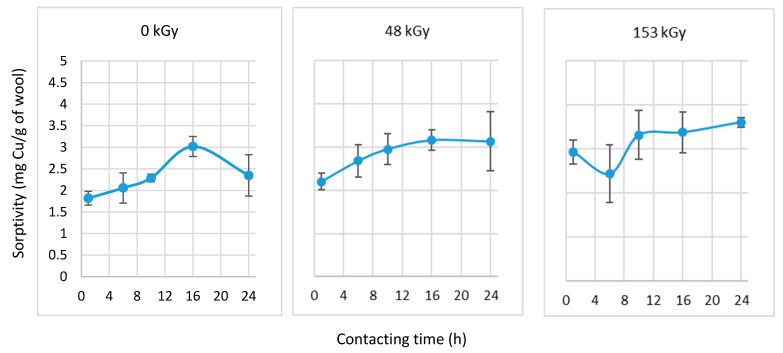
Sorptivity after the selected prewetting interval and 1 h contact with CuSO_4_ solution (6 mM).

Figure 4 and Figure 5 show that each prewetted sample exhibited increased sorptivity within contact times less than 24 h compared to the non-prewetted samples (Figure 4). The sorptivity of each sample showed a specific development with dose and time. However, the mechanism of the corresponding process requires more in-depth evaluation. If we were to consider the extreme values (Figure 5), the highest sorptivity was observed in the 48 kGy-dosed sample after 5 h of contact with the solute. However, it would be interesting to determine which prewetting duration would be technologically acceptable to allow a minimum of 1 h of contact with the solute, thereby speeding up the adsorptive separation. The results of such an experiment are shown in Figure 6.

**Figure 7:**
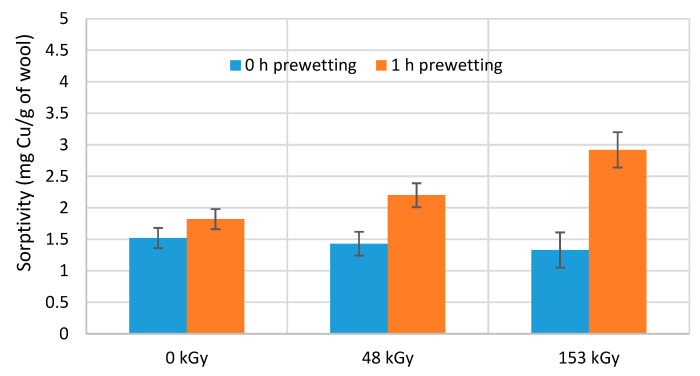
Sorptivity of wool samples non-prewetted and 1 h prewetted followed by 1 h contact with CuSO_4_ solution (6 mM).

Prewetting the wool positively affected sorption, with an increasing dose improving the sorptivity. Since the shortest possible duration of the individual steps is advantageous for the practical application of the adsorption process, we assessed the sorption after 1-h prewetting and subsequent 1-h contact. The results were compared to samples with the same contact time without prewetting (Figure 7).

**Figure 8:**
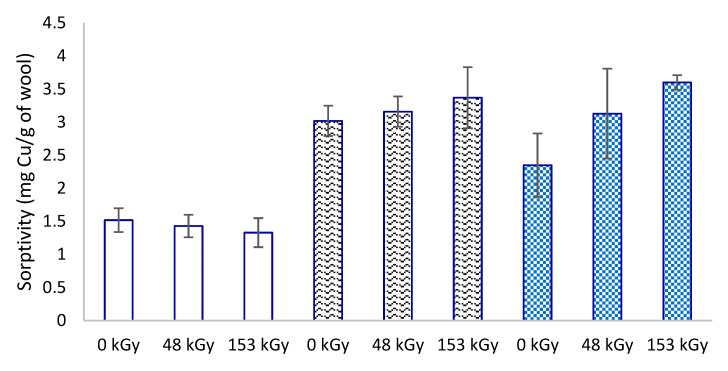
Sorptivity of non-prewetted wool samples (empty columns) and after 16 h (wave pattern) and 24 h (checkerboard) prewetting followed by 1 h contact with CuSO_4_ solution (6 mM).

Increasing the absorbed dose for non-prewetted samples after 1 h of contact reduced the sorptivity, although the decrease did not appear statistically significant (Figure 7). Meanwhile, the same samples after 1 h of immersion and the same contact time consistently exhibited higher sorption, increasing with the absorbed dose (Figure 7). Such a contradictory observation may be perplexing at first glance. Indeed, under certain experimental conditions, the sorptivity trend changes unexpectedly, particularly for prewetted samples (Figure 5 and Figure 6), and is likely related to the interaction of the S-oxidized wool products with water. Although the sorptivity of each sample culminates differently after 1 h of contact with Cu(II), from the dependences in Figure 6, it can be deduced that 16 h of prewetting provides the highest sorptivity for most samples. Therefore, we compared the non-prewetted samples and the samples with longer prewetting for 16 and 24 h followed by the same 1 h contact with the solute (Figure 8). Here, the beneficial effect of the prewetting was even more evident. The results also confirmed that the wool irradiation improved its sorption properties for the selected solute. Prewetting for 24 h compared to the corresponding non-prewetted sample after 1 h contact increased the sorptivity for 0 kGy by 55%, 48 kGy by 120%, and 153 kGy up to 170%.

In the adsorption isotherm fitting test, chemisorption was confirmed in the case of Cu adsorption by applying the Flory-Huggins model [29]. In chemisorption, the Cu(II) cation reacts with the R-COOH groups on the keratin side chains (such as aspartic acid, glutamic acid, and cysteine) to form Cu-salts. Cu(II) readily forms complex structures with ligands originating from keratin (=NH, -NH_2_, -OH, -SH) [12] and with a high probability from neighboring chains. This creates crosslinking, making it difficult for further Cu(II) ions to diffuse to the active adsorption sites in the bulk. Meanwhile, cooperative adsorption (or binding) occurs when lateral interactions between molecules on the active surface participate [30]. In the case of wool, the surface provides not only carboxyl or cysteic acids (main active points) to form salts with the cations but also donor groups. These form coordinative bonds to create corresponding complex salts, which present the cooperative binding. Such processes take place in non-irradiated and irradiated wools. However, in the irradiated wool, due to the transformation of disulfide bridges to the final cysteic acid (Scheme 1), complex Cu(II)-cysteinates are added to the complex Cu(II)-carboxylates. Therefore, although crosslinking is more frequent in the irradiated samples and slows down diffusion, its effect is compensated by increased Cu(II) bound as cysteinate. The rate of these actions is a function of the absorbed dose. During wool prewetting, water diffuses through the membrane more easily thanks to the smaller molecules and binds to potential adsorption sites via H-bonds. Thus, it provides more favorable conditions for the subsequent displacement of water by the solute, although non-equivalent [28]. A smaller surface tension difference between the solute solution and water than between the solute and wool can favorably contribute to water displacement (Figure 3). In the case of Cu(II), a chemisorption mechanism facilitates solute binding to wool. This process occurs until an equilibrium is reached, which is likely specific to each solute and the conditions, including concentration and spatial possibilities.

Figure 8 shows that 16 h prewetting of the non-irradiated wool (0 kGy) led to higher sorptivity than 24 h prewetting. We believe that during the 24-h prewetting, further formation and possibly mutual transformation of unidentified associates continued; the interaction of these associates with the active points of keratin in the solution were hindered, resulting in lower sorptivity. However, the sorption processes of the different samples cannot be fully defined due to differences in the structures generated by the effect of the electron beam.

In summary, the results of the sorption experiments with a low-concentration Cu(II) solution (6 mM) show that the prewetting of natural and electron beam-irradiated sheep wool improves the efficiency of Cu(II) sorption.

### 3.2. Testing of Wool Prewetting Using Cu(II) Adsorption from 50 mM-Solution

In a previous related study on the adsorption of Cu(II) on wool from solutions with a higher concentration of Cu(II) (10–80 mM) [12], a significant fluctuation in sorption with absorbed dose and concentration was reported. Based on FTIR analysis, the fluctuation was attributed to the variable complexation ability of copper and the structure of complexes with ligands provided by keratin. Therefore, for comparison, we performed analogous sorption experiments with solutions containing a higher concentration of Cu(II).

#### 3.2.1. Non-Prewetted Sheep Wool

**Figure 9:**
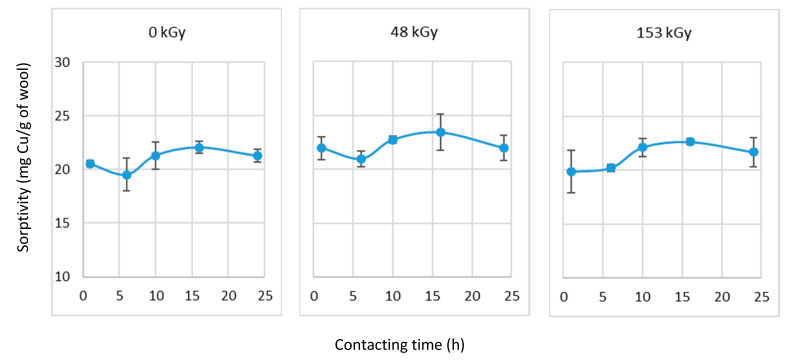
Dependence of non-prewetted wool sorptivity on contact time with CuSO_4_ solution (50 mM).

A significantly higher sorption from the 50 mM Cu(II) solution than the 6 mM solution is reasonable due to the different concentrations. The sorption results of the non-prewetted samples are shown in Figure 9.

The differences in the sorptivity of the non-prewetted samples were not statistically significant, with an average sorptivity of 21.6 mg/g. The results qualitatively replicate the order of sorption from the 6 mM Cu(II) solution after 24 h of contact (Figure 4) in the sample order of 48 kGy > 153 kGy ≈ 0 kGy with an average of 2.5 mg/g. This replicative result in the irradiated samples was due to the addition of cysteic acid as a binding site in contrast to the 0 kGy sample, which exhibited the lowest sorptivity. Despite the higher cysteic acid content in the 153 kGy samples than the 48 kGy, the higher density of the formed crosslinks serves as a greater obstacle to the diffusion of Cu(II) into the matrix bulk, lowering the sorptivity.

#### 3.2.2. Prewetted Sheep Wool

**Figure 10:**
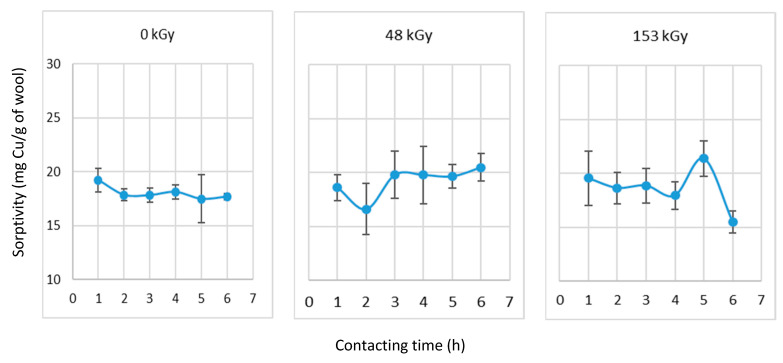
Sorptivity of wool after 24 h of prewetting at selected contact times with CuSO_4_ solution (50 mM).

The samples with each absorbed dose were subjected to 24 h prewetting followed by contact with 50 mM Cu-solution for 1–6 h, similar to the adsorption from the 6 mM solution (Figure 5). The sorptivity results under these conditions are shown in Figure 10.

Although the sorptivity of 24-h wetted non-irradiated wool of the 0 kGy was relatively the same for all selected contact times with an average of 18 mg/g, considering the dispersed results, an overall decrease occurred compared to the initial value. The average value was lower than the sorptivity of 21.3 mg/g determined for the non-prewetted wool after 24 h of contact with the Cu(II) solution (Figure 9). Since the 0 kGy wool prewetted for 24 h (Figure 10) did not exhibit increased sorptivity with extended contact, but rather the opposite, in this case, the prewetting had no effect. Strictly speaking, 1 h of contact was determined to be the most appropriate. A similar conclusion is also acceptable for the sorptivity of the 48 kGy-dosed wool. That is, the average sorptivity of the prewetted 48 kGy wool was 19.4 mg/g for the applied contact times without significant differences (Figure 10), while the sorptivity of the non-prewetted wool was 22 mg/g (Figure 9). In contrast, the prewetted wool with the 153 kGy dose exhibited different values (Figure 10); the sorptivity in the 1–4 h contact interval did not differ from the 18.7 mg/g; however, after 5 h, a maximum of 21.4 mg/g was achieved and after 6 h a minimum of 15.5 mg/g. The non-prewetted 153 kGy sample after 24 h of contact showed a sorptivity of 21.7 mg/g (Figure 9). These results indicate that complex restructuring time-dependent actions occurred that measurably affect the sorptivity. At a higher solute concentration, a denser network of variable Cu-complexes will form on the surface of keratin helixes; this will hinder the diffusion of other solute molecules, limiting the displacement of water from the vacuoles coming from the prewetting. A larger difference between the surface tension of a more concentrated solution and wool can also contribute to the lower exchangeability of copper for water (Figure 3). However, a more in-depth analysis of this hypothesis is needed. 

**Figure 11:**
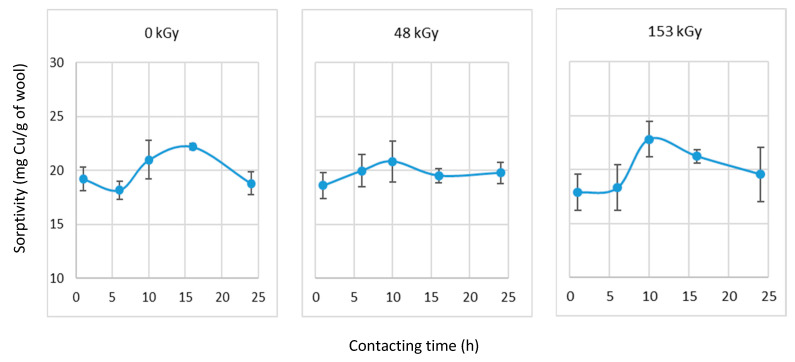
Sorptivity after selected prewetting interval and 1 h contact with the CuSO_4_ solution (50 mM).

Next, we evaluated the combined influence of a prewetting time < 24 h with 1 h of contact. The results are shown in Figure 11.

**Figure 12:**
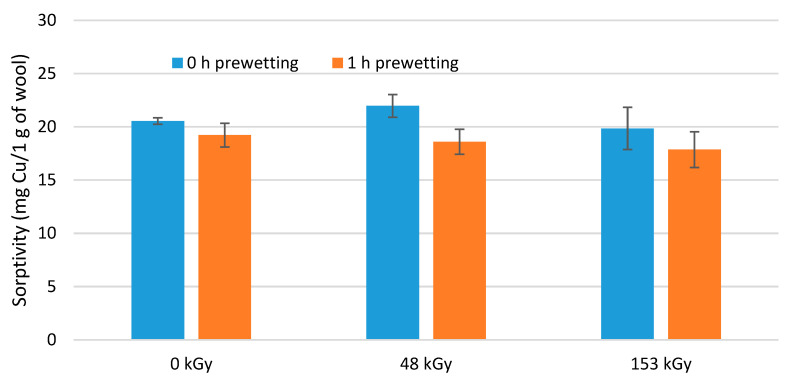
Sorptivity of wool samples non-prewetted and 1 h prewetted followed by 1 h contact with a CuSO_4_ solution (50 mM).

From the 0 kGy sample results, 16 h prewetting appeared to be the most favorable; for both irradiated samples the highest sorptivity was achieved after 10 h of prewetting (Figure 11). Considering the variance in the results, 10 h of prewetting would be acceptable for all samples. The most economical conditions of the separation process in this case would be the non-prewetted samples or prewetted for 1 h with 1 h Cu(II) contact. The data are illustrated in Figure 12 and are designed in parallel to Figure 7. However, comparing the results leads to different conclusions.

While at a low Cu-solute concentration of 6 mM, 1 h prewetting proved to be beneficial for samples with a 1-h contact (Figure 7), a higher solute concentration of 50 mM under the same conditions led to the opposite conclusion, namely that the prewetting is not significant as it reduces the sorptivity (Figure 12). The sorptivity of the non-prewetted wool with a long contact time of 24 h provided relatively the same results as with 1 h of contact. This would correspond to the aforementioned hypothetical adsorption mechanism, in which the adsorbate cooperatively binds immediately to the surface of the adsorbent, saturating it and creating a barrier for further sorbate to enter despite longer contact times. Although there are pores in the matrix of the adsorbent (Figure 2) and thus the conditions for physisorption, the solute cannot readily reach them. Even if, during prewetting, water first occupies the active sites, the sorbate will not displace it quantitatively, thus reducing the sorptivity of the wool for the given solute.

Thus, the sorption experiments with a Cu(II) solution with a higher concentration (50 mM) imply that, under such conditions, prewetting both natural and electron beam irradiated sheep wool does not significantly affect the sorption efficiency.

## 4. Conclusions

The batch method of Cu(II) sorption on sheep wool, both natural and irradiated with an accelerated electron beam, was used to examine the effect of wool prewetting and solute contact time with the sorbent on the sorption result. Experiments included prewetting of the samples for 0 to 24 h, contact between the Cu(II) solute and wool for 1 to 24 h, and selected combinations of these parameters. The CuSO_4_ solutions with different concentrations, namely 6 mM and 50 mM, were used for the study. The sorption results showed that wetting the wool before sorption from a less concentrated solution is important and can improve efficiency and accelerate the separation process. On the contrary, when applying a more concentrated solution, the prewetting slightly reduced the sorptivity. The reason is considered to be the cooperative adsorption of Cu(II)-carboxylates and cysteinates with the formation of a crosslink through keratin ligands already on the surface of the fiber, creating a barrier for diffusion of the solute into the wool block. The displacement of the prewetting water with the solute in the internal active sites is thus made more difficult and leads to lower sorption of Cu(II). The obtained results imply that the acceleration of sorption of some complexing solute on prewetted wool is concentration-dependent. This hypothesis should be supported by the study of the sorption of some substance with no tendency to form a complex, where it can be expected that the prewetting of the wool will be useful even at a higher concentration of the solute. This anticipation will be the subject of further investigation. The knowledge acquired in the present work serves as a basis for the optimization of analogous adsorption processes in technological practice using electron-modified wool.

## Data Availability

Not applicable.
